# A comparative study on the effects of different cryoprotectants on the quality of canine sperm during vitrification process

**Published:** 2016-09-15

**Authors:** Sahar Nouri Gharajelar, Rajab Ali Sadrkhanloo, Masoud Onsori, Adel Saberivand

**Affiliations:** 1 *Department of Basic Sciences, Faculty of Veterinary Medicine, Urmia University, Urmia, Iran;*; 2 *Private Veterinary Practitioner, Urmia, Iran;*; 3 *Department of Clinical Sciences, Faculty of Veterinary Medicine, University of Tabriz, Tabriz, Iran.*

**Keywords:** Cryoprotectant, Dog, Sperm, Vitrification

## Abstract

Cryopreservation has the capacity to extend spermatozoa’s lifespan and viability. In addition, the semen samples can be collected, preserved and stored or sent to distant locations and still be used long after the death of the semen donor. In this study for the vitrification of dog sperm (fresh and swum-up sperm), different cryopreservation mediums on the basis of glycerol, milk and egg yolk were used. Then, all of the samples were vitrified in the liquid nitrogen and thawed at least 48 hr later for re-examination of sperm parameters. The sperm parameters before and after cryopreservation in all groups were compared. It was found that during vitrification process, spermatozoa were damaged by the mechanical blows in centrifugation during swim-up processing, so they had less resistance than fresh semen. The examination of different cryoprotectants revealed that milk has better effects on the cryopreservation of semen than glycerol and egg yolk. With the comparison of the effects of glycerol and egg yolk as cryoprotectants, it was found that glycerol had better effects than egg yolk on the cryopreservation of the semen. In conclusion, milk might be used as a cryoprotectant instead of glycerol for canine sperm cryopreservation.

## Introduction

Cryopreservation of dog semen is widely used for long term preservation of genetic materials or transporting it among countries.^1^ Artificial insemination with preserved semen collected from dogs of documented high quality provides the breeders with a good tool for widening the desired gene pool.^2^

The addition of cryoprotectors to semen extenders improves cell survival after the freezing process. Cryoprotectors are divided in to two groups. First are intracellular cryoprotective agents like glycerol and dimethylsulfoxide. Second are extracellular ones such as proteins and sugars. Glycerol is the cryoprotector often used to freeze semen of different species.^3^ As a permeable cryoprotectant, glycerol prevents the formation of ice crystals inside the cells. However, it has been reported that this cryoprotectant has toxic effects on spermatozoa, such as physicochemical alterations that can lead to rupture of the plasma membrane or removal of important membrane proteins, and cause acrosomal damage, which will be reflected in reduced fertility. Its toxic effects on sperm have stimulated the study of other cryoprotectors.^4^^,^^5^

The ideal cryoprotector should have a low molecular weight with high water solubility and low toxicity.^6^ However, there is a great diversity in cryobiological responses of different cell types or given cells among different mammalian species. Cryosurvival requires that cell freezing and thawing be done within certain biophysical and biological limits defined by the following cryobiology principles: cells should be frozen in such a way that little or none of their intracellular water freezes. They should be warmed in such a way that unfrozen intracellular water remains unfrozen during warming, or that small ice crystals formed during cooling remain small during warming. Even the aforementioned conditions are important; most cells will not survive unless substantial concentrations of cryoprotectors are used.

These factors and mechanisms (or hypotheses) of cryoinjury and its prevention are discussed, including the most famous "two factor hypothesis" theory of Peter Mazur, concepts of cold shock, vitrification, cryoprotective agents (CPAs), lethal intracellular ice formation and osmotic injury during the addition/removal of CPAs and during the cooling/warming process as well as modeling/methods in the cryobiological research.^7^

It is well known that milk and egg yolk are non-permeable cryoprotectants. They prevent formation of ice crystals outside the cells, provide enough energy for the spermatozoa and also play the role of phosphate buffer for them.^8^

The objective of the present study was to compare the effects of milk, egg yolk and glycerol as cryo-protectants on canine semen quality after vitrification and thawing processes.

## Materials and Methods

Animals and semen collection. Ten sexually mature German shepherd dogs aged from 2 to 6 years, and owned by private clients were used. The ejaculates of all dogs were collected by means of digital stimulation following a period of 3 to 5 days of sexual abstinence.^9^

Semen evaluation. Semen volume, pH, viscosity and color were macroscopically evaluated. Motility (percentage of moving spermatozoa) and vigor (type of motility) were determined in semen using light microscopy through counting 100 cells per slide. Sperms with amorphous head, small head, large head, double head and cytoplasmic droplet were considered to have body defects and sperms with double tail, coil tail and blunt tail were considered to have tail defects.^10^ Sperm concentration was determined by Neubauer counting chamber (Sigma Aldrich, Steinheim, Germany). Only samples that presented a volume ≥ 0.60 mL, concentration > 200 × 10^6^ spermatozoa per mL, motility ≥ 80% and vigor ≥ 4 were used in the experiment.^11^

Sperm washing and swim-up methods. After centrifuging of entire ejaculates for 10 min at 200 g, the supernatant was discarded immediately and the pellet was overlaid with 0.30 mL Ham’s F_10_ medium (Sigma Aldrich) and incubated for about 60 min at 37 ˚C. A quantity of 0.20 mL of the supernatant was drawn up into the tube, and then its motility, vigor and viability were assessed.^12^

Extension of semen. The extender consisting of 3.02 g tris-hydroxymethyl-aminomethane (Sigma Aldrich), 1.78 g monohydrated citric acid (Sigma Aldrich) and 1.25 g D-fructose (Sigma Aldrich), dissolved in 100 mL of distilled water was used.^3^ Then, this solution was divided into two equal 50 mL portions. Ten percent of the first was then replaced by egg-yolk and 10 percent of the second was replaced by milk. The solution one was divided into two equal portions which were named 1a and 1b. The solution 1b should contain 12% glycerol, therefore, 3 mL of this solution were replaced by glycerol (Sigma Aldrich).

Freezing process. The freezing process was done by adding one portion of the extender to one portion of semen (fresh and swum up samples) at 37 ˚C immediately after initial analysis.^2^ The equilibration period was elapsed after 10 min. Then, samples were frozen in 0.25 mL straws 2 cm over – 70 ˚C liquid nitrogen vapor for 10 min before being plunged into liquid nitrogen at – 196 ˚C. Straws were stored in liquid nitrogen for at least 48 hr before thawing. Semen straws were thawed in a 38 ˚C water bath for 60 sec. After thawing, sperm motility, vigor and viability were evaluated in all samples.^13^

Statistical analysis. The effects of vitrification on sperm motility and morphology by using three different cryoprotectants were evaluated by ANOVA and Tukey’s tests using SPSS (version 22, SPSS Inc., Chicago, USA). The results were expressed as mean and standard deviation. The results were considered significant when p < 0.05.

## Results

The fresh semen of dogs was white in color with a milky viscosity. All of the samples were normospermia. Other characteristics are summarized in Table 1.

 After vitrification of semen samples using the examined cryoprotectants, it was seen that swum-up samples were damaged by mechanical blows during swim-up processing, so they could not well bear vitrification process and most of the spermatozoa in swum-up samples were died. But, vitrification by itself reduces the quality of semen samples.

**Table 1:** Means and standard deviations of the characteristics of fresh semen obtained from dogs (n = 10).

**Semen Characteristics**	**Mean ± SD**
**Semen volume (mL)**	1.55 ± 0.49
**pH**	7.20 ± 0.02
**Total motility (%)**	9.05 ± 2.83
**Progressive motility (%)**	76.50 ± 7.47
**Fair motility (%)**	11.50 ± 4.74
**Poor motility (%)**	2.50 ± 3.53
**Alive sperms (%)**	90.50 ± 2.83
**Dead sperms (%)**	9.52 ± 0.83

Table 2 shows the effects of different cryoprotectants on the parameters of centrifuged-derived vitrified and swum-up samples. There were significant (*p* < 0.05) differences in the percentages of total motility and alive spermatozoa. For determining the differences between groups, Tukey test was used. Total motility and percentage of alive spermatozoa were significantly higher in milk added centrifuged-derived vitrified samples than glycerol added centrifuged-derived vitrified ones (*p* < 0.05), (Table2). Also, percentages of total motility and alive spermatozoa were significantly higher in glycerol added centrifuged-derived vitrified samples than egg yolk added centrifuged-derived vitrified ones (*p* < 0.05), (Table2).

Milk added vitrified centrifuged-derived samples had significantly higher total motility and percentage of live spermatozoa than egg yolk added vitrified centrifuged-derived samples (Table 2). Percentages of total motility and alive spermatozoa were significantly higher in glycerol added vitrified fresh samples than glycerol added vitrified swum-up ones (*p* < 0.05), (Table 2). Between milk vitrified centrifuged-derived samples and milk added vitrified swum-up ones, there were no significant differences in the semen parameters (*p *< 0.05).

**Table 2:** Means and standard deviations of motility, viability and body and tail defects percentages of centrifuged-vitrified-sperms and swam up-vitrified-sperms extended in glycerol, milk and egg yolk in dogs

**Parameters**	**Centrifuged sperm**				**Swum-up**		
	**Milk**	**Egg yolk**	**Glycerol**		**Milk**	**Egg yolk**	**Glycerol**
**Motility**	79.50 ± 5.98^a^*	53.00 ± 9.48^b^	66.00 ± 8.09^c^*		69.50 ± 7.61^a^*	65.00 ± 7.45^b^	53.00 ± 9.48^c^*
**Viability**	79.50 ± 5.98^a^*	53.00 ± 9.48^b^	66.00 ± 8.09^c^*		69.50 ± 7.45^a^*	65.00 ± 12.15^b^	53.00 ± 7.61^c^*
**Body defects**	3.20 ± 1.32	3.50 ± 1.50	4.50 ± 1.58		4.30 ± 1.66	6.40 ± 1.97	3.50 ± 1.50
**Tail defects**	16.50 ± 5.93	23.60 ± 8.59	17.50 ± 5.91		19.70 ± 5.92	19.60 ± 6.14	24.10 ± 8.50

abc Different superscript letters in each row indicate significant differences among groups.

* indicates significant difference among centrifuged and swum-up groups (*p *< 0.05).

Percentages of total motility and alive spermatozoa were significantly higher in egg yolk added vitrified centrifuged-derived samples than egg yolk added vitrified swum-up ones (*p* < 0.05), (Table 2). 

The present study showed that spermatozoa vitrified with milk as an extender, have better quality than spermatozoa vitrified with glycerol and egg yolk. Comparing glycerol and egg yolk as cryoprotectants, glycerol was better than egg yolk. 

## Discussion

In this experiment, three different cryoprotectants were used during vitrification process. Glycerol acts as a permeable cryoprotectant but milk and egg yolk act as non-permeable cryoprotectants. Milk and egg yolk buffer spermatozoa and act as energy sources.^8^ To avoid excessive cell shrinkage during slow cooling, permeable and non-permeable cryoprotectants have been used.^14^ We used glycerol as a permeable cryoprotectant to prevent extracellular ice crystals formation, providing enough energy for the spermatozoa and also to provide adequate phosphate buffer. Milk and egg yolk were used as non-permeable cryoprotectants.

Vitrification of sperm is an important routine technique used in the management of male infertility in many species. Decrease in spermatozoa motility after cryopreservation is an important fact.^14^ Cell injury during vitrification is caused by formation of intracellular or extracellular ice crystals and osmotic damages. Thawing of the cells can also deteriorate viability through possible excessive osmotic swelling.^15^^,^^16^ Slow freezing can also cause extensive chemical and physical damages to sperm cell membranes due to change in lipid-phase transition or increased lipid peroxidation. It is well known that production of reactive oxygen species leads to an increase in lipid peroxidation after cryopreservation;^16^ and this event is correlated with a loss of sperm motility.^17^ As a result, the percentage of motile spermatozoa decreases after vitrification. In this study, in order to eliminate the damages to the sperm due to slow freezing process, we used –70 ˚C liquid nitrogen vapor and liquid nitrogen at –196 ˚C.

Previously, the effects of single and fractionated glycerol addition on canine semen quality after thawing have been compared. It has been concluded that glycerol can be added to canine semen in single or fractionated manner, but the single addition method is the easiest and the most practical to use.^3^ Here, we also used the single addition method for the cryoprotectants used.

Further, the cryopreservation of canine semen using a coconut water extender with egg yolk and three different glycerol concentrations has been studied. It was found that three glycerol concentrations can be used successfully in cryopreservation of canine semen using a coconut water extender.^2^

Since glycerol has detrimental effects on the fertility of fresh cooled semen and its toxic effects on sperm have been reported by many authors, the study of other cryoprotectants is needed.^6^

 Effect of adding various concentrations of dimethyl formamide on characteristics of canine semen diluted in powdered coconut water has been shown.^18^ Based on concentrations, dimethylformamide together with ACP-106C (ACP Biotecnologia, Fortaleza, Brazil) and 10% egg yolk as diluents, yielded unsatisfactory *in vitro* results for freezing canine semen. Amides belong to the group of penetrating cryoprotectants, which decrease intracellular freezing point by means of their colligative properties. Thus, more water will remain in a liquid state in low temperatures, decreasing the intracellular concentration of solutes and creating a less harmful environment for sperm during freezing.^18^

The ability of sucrose to protect spermatozoa against mitochondrial damage, artificial acrosome reaction and DNA fragmentation during ultra-rapid cryopreservation in canine sperm has been investigated.^19^ It has been concluded that sucrose, a non-permeable cryoprotectant, can effectively preserve important physiological para-meters such as mitochondrial membrane potential and DNA integrity during ultra-rapid cryopreservation.^19^

Egg yolk is a common part of semen diluents which protects the spermatozoa against cold shock and confers protection during freezing and thawing. It is believed to act at the level of the cell membrane.^19^ Our results were also consistent with these reports.

Milk has been used for freezing semen mostly in reconstituted form, combined with arabinose, fructose or egg yolk. In a comparative laboratory study, it was found that pasteurized whole or reconstituted skim milk, citrate-yolk, milk-glucose and citrate-glucose-yolk are of equal value.^19^ In accordance with our results, others reported better post-thaw sperm survival after freezing with reconstituted skim milk than citrate-yolk, fructose or lactose based diluents.^19^ Addition of egg yolk to heated homogenized milk did not increase post-thaw sperm survival.^20^

In conclusion, as a canine semen cryoprotectant, milk acts better than glycerol and egg yolk. Also, glycerol acts better than egg yolk during vitrification process.
